# Alloy information helps prioritize material criticality lists

**DOI:** 10.1038/s41467-021-27829-w

**Published:** 2022-01-10

**Authors:** T. E. Graedel, Barbara K. Reck, Alessio Miatto

**Affiliations:** Center for Industrial Ecology, School of the Environment, Yale University, 195 Prospect St, New Haven, Connecticut 06511 United States

**Keywords:** Materials science, Sustainability

## Abstract

Materials scientists employ metals and alloys that involve most of the periodic table. Nonetheless, materials scientists rarely take material criticality and reuse potential into account. In this work, we expand upon lists of “critical materials” generated by national and regional governments by showing that many materials are employed predominantly as alloying elements, which can be a deterrent to recovery and reuse at end of product life and, likely as a consequence, have low functional end-of-life recycling rates, among other problematic characteristics. We thereby single out six metals for enhanced concern: dysprosium, samarium, vanadium, niobium, tellurium, and gallium. From that perspective, the use of critical metals in low concentrations in alloys unlikely to be routinely recycled should be avoided if possible. If not, provision should be made for better identification and more efficient recycling so that materials designated as critical can have increased potential for more than a single functional use.

## Abstract (web summary)

Critically lists tend to ignore the fact that most metals are largely used in alloy form. Here the authors analyze four key metrics and show that six critical metals can be singled out for enhanced concern – Dy, Sm, V, Nb, Te, and Ga.

## Introduction

In 2008, a committee of the U.S. National Academies published an analysis of which nonfuel minerals might be termed “critical” to the national economy^1^. In that work, the study identified eleven minerals or mineral groups as having at least some degree of criticality. Perhaps more important, the committee devised a two-axis rating system based on supply risk and impact of supply disruption. Two years later, the basic idea was utilized (with some revisions) in determinations of criticality for the European Commission^2^ and the U.S. Department of Energy^3^. Since that time, several different approaches to critical materials’ assessment and response have been devised^4–8^. The unstated implication of those efforts is that they apply to the relatively short term, certainly less than a decade. However, no general methodological coherence has resulted.

**Fig. 1:**
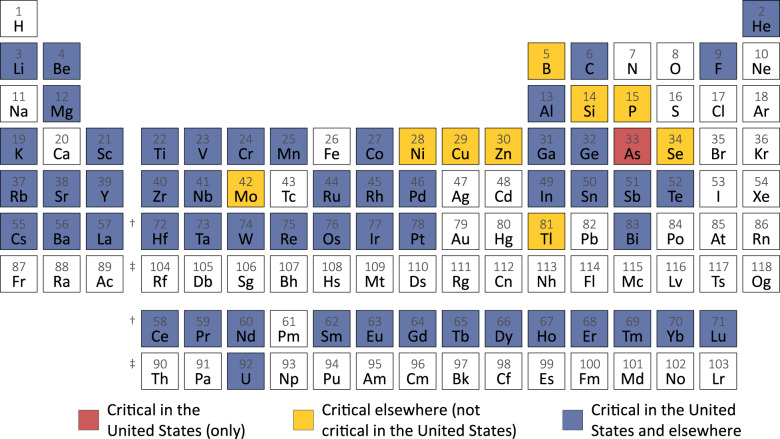
Elements designated critical only by the U.S. Department of the Interior 11, only by other countries than the United States (European Union 9, Japan 14, Australia 12, and Canada 13), and by both the United States and other countries.

Nonetheless, governments have continued to develop their own methodologies and to generate critical material lists. Perhaps, the most widely known and best described are those of the European Commission^9^ and the United States^10,11^. The European Commission currently designates twenty-six materials (mostly metals) and three metal groups as critical, while the U.S. list comprises thirty-two materials (mostly metals) and two metal groups. In addition, Australia, Canada, and Japan recently published their own criticality lists^12–14^. None of the lists from these five authorities include the very abundant and nonmetal alloy forming hydrogen, nitrogen, oxygen, calcium, halogens (except fluorine), or noble gases. All lists differ to some degree, so the total number of materials identified is larger than it might appear from a cursory glance at the individual lists. We display the integrated criticality results in Fig. 1.

It is interesting that rather than asking what is critical, it might be more appropriate to ask which metals or metalloids (the alloy formers) do not appear in any of the above lists. It turns out that there are only six: iron, silver, cadmium, gold, mercury, and lead. The lack of concern relates to the fact that iron, silver, and gold have long histories of mining and recycling because of their broad importance in technological utility and economic value, while cadmium, mercury, and lead are disfavored as a consequence of their high levels of toxicity. (Note that because much of the discussion in this paper is focused on alloys, the term “metal” will be used hereafter to encompass metals, metalloids, and other alloying elements.)

One important aspect of the National Academies report^1^ that has seen little recognition in the decade since its publication is the demonstration (cf. figure 4.3 and adjoining discussion in the report) that materials in different metallic forms and diverse final sector uses could be regarded as having several criticality designations. This situation results in part because certain end uses are regarded as more important or less substitutable than others, and also because the forms of elements in their various uses are often different, particularly in that metal use is often in alloy forms and because alloying and other materials’ mixing can sometimes frustrate end-of-life recycling^15^. Alloys generally consist of a dominant (major) metal and small percentages of other metals (minor alloying elements) that are added to increase material performance in one way or another^16^. Because alloy compositions are designed to meet detailed performance requirements for specific products, the number of tested and marketed alloy compositions runs into the thousands. This material complexity has generally deterred criticality analysts from venturing into studies of alloy production, use, recycling, and loss; nonetheless, alloy complexity has important but largely unexplored consequences for criticality assessment. For example, it is virtually impossible to create a recycling system that handles separately thousands of alloys without mixing of the respective grades, leading to unwanted changes in alloy compositions.

Many modern alloys incorporate five or more elemental constituents^17,18^. Examples include shape-memory alloys, self-healing alloys, bulk metallic glasses, high-entropy alloys, and advanced thermoelectrics (Table 1). Note that not one of the metal groupings in Table 1 avoids metals characterized as critical; some (e.g., HSLA steels, bulk metallic glasses) include many critical metals; indeed, few of the groups contain elements that are not designated critical by one governmental entity or other. This recent increase in material complexity would seem to invite consideration of criticality by materials’ designers. Nonetheless, a current commentary on alloy design^19^ avoids the topic completely, demonstrating the insufficient communication that exists among the respective research communities.

**Table 1:** Examples of the diverse compositions of many modern alloys.

Group designation	Metals typically included	References
Stainless steels	Fe, *Ni*, **Cr**, **Mn**, *Mo*	Reardon^50^, International Stainless Steel Forum^51^
High-strength low alloy steels	Fe, **V**, **Cr**, **Mn**, **Nb**, *Mo*	Davis^52^, Kuziak, Kawalla^53^
Nickel superalloys	*Ni*, **Al**, **Cr**, **Co**, *Mo*, **Hf**, **W**, **Re**	Pollock and Tin^17^, Long, Mao^54^
Bulk metallic glasses	**Zr**, **Hf**, **V**, **Ti**, **Be**	Kruzic^55^, Mota, Graedel^56^
High-entropy alloys	**Al**, **Cr**, **Co**, *Cu*, Fe	Tsai and Yeh^57^, George, Raabe^58^
Advanced thermoelectrics	Pb, **Te**, *Se*, **Sb**, **Ge**	Gaultois, Sparks^59^, He and Tritt^60^
Shape-memory alloys	**Ti**, *Ni*, *Cu*	Mohd Jani, Leary^61^, Shelyakov, Sitnikov^62^
Self-healing alloys	**Al**, *Cu*, **Mg**	Ferguson, Schultz^63^, van Dijk and van der Zwaag^64^

Notes: Metals in bold face are deemed critical by both the United States^11^ and at least one other governmental authority^9,12–14^. Those deemed critical by at least one government except the United States are in italics.

A related aspect of the use of complex alloys is that recycling and reuse of many of these alloys “requires a sophisticated and agile metallurgical infrastructure”^20^, with some of the alloy metals being lost to slags, slime, or speiss rather than being recoverable in current technology.

It is important to recognize that, in general, the minor alloying elements are not mined for themselves (rhenium, for example), but rather as low-concentration “companions” in the ores of major metals. Consequently, the supplies of minor alloying elements can be rather unresponsive to rapid changes in demand. The availability of specific alloys may thus be constrained even when the major metal is widely available. A criticality analysis that ignores this material complexity is not taking advantage of all of the supply and demand information and the resulting implications that could be incorporated.

Another criticality topic worth mentioning is the common perception that if critical metals are unavailable in desired quantities, a suitable substitute can be employed. However, detailed analyses^21^ and references therein^22^ indicate that no satisfactory substitute exists for many uses of critical metals, or that employing a substitute will result in significant price and performance penalties (e.g.,^23^). Performance degradation upon substitution is particularly likely in the case of alloys whose compositions aim at very specific physical and chemical properties^24^.

A final aspect of alloys that is of broad interest for corporate and national policy is that individual minor alloying elements are sometimes designated as “critical” by industrial or governmental groups^5,6,25,26^ as well as by governments. These criticality designations have been made for elements (or groups of elements such as “heavy rare earths”), not for alloys, but the life cycles of many minor alloying elements are often defined by their use in alloy form.

In this work, we study the current state and implications of critical materials in alloys, especially as regards losses or nonfunctional recycling (i.e., recycling after which alloying elements are present in the new material as “tramp elements” that serve no useful purpose and may in some cases be detrimental^27^). Alloys contain multiple metals, many of which are considered critical and account for only small fractions of the total. The process of sorting and collecting alloyed products in waste streams does not consider—on most occasions—specific alloys. It tends to lump together alloys whose main alloying element is the same (e.g., steels are all considered one big category, disregarding the specificities of each alloy). This research aims to generate data on the fractional alloy use for metallic elements of the periodic table and single out the critical elements that are routinely used in alloy form and that are not recycled. To do so, we collect data on use in alloy form, end-of-life losses, mining companionality, and import dependency for 69 elements of the periodic table. The results indicate that six elements, dysprosium, samarium, vanadium, niobium, tellurium, and gallium, are almost entirely used as alloying elements and are nearly never functionally recycled. These findings should point material scientists, product designers, and policymakers toward alternative materials and promote better recycling policies for the alloys in which these materials are involved.

## Results

**Fig. 2:**
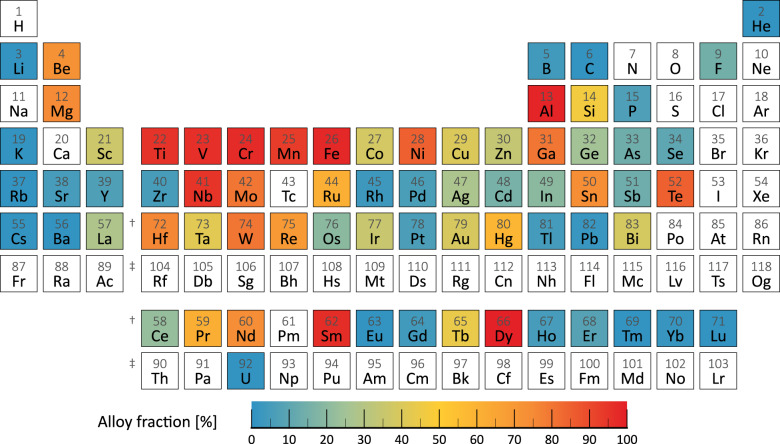
Fractional use of elements in alloy form. Estimates on the alloy use per end use sector and element are provided in Supplementary Table 3.

The degree to which metals are used in alloy form, displayed in Fig. 2, provides a starting point for the work discussed in this paper.

The alloy fraction has not traditionally been chronicled for metals, but it can be estimated from studies of the principal fractional uses of the metals. Drawing upon information from the European Commission^9,22^, Schulz et al.^10^, Ciacci et al.^15^, and other studies (cf. Supplementary Table 3), Fig. 2 presents a “periodic table of alloy fractions”. It turns out that for twenty three of the metals more than half of metal demand is alloy-related. This can be the case for alloy host metals such as iron or nickel, but also for metals employed in small concentrations within large alloy flows (e.g., beryllium in copper electronic connector materials). In other cases (selenium, silver, antimony, tantalum, etc.) the alloy fraction is less than 50% but still significant from the perspective of important properties such as embodied energy and recycling potential^28^. Further information and literature citations for individual metals are available in the Supplementary Information (Supplementary Table 3) and in the Source Data.

**Fig. 3:**
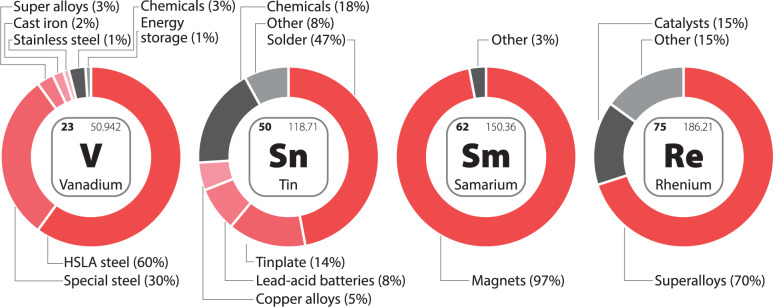
Final uses of vanadium, tin, samarium, and rhenium circa 2020. Shades of red indicate the use in alloy form.

The major uses of metals are not stable year to year, but constantly evolve as research provides new opportunities for the employment of metals and as markets wax and wane. Nonetheless, the major uses tend to change slowly over time, and thus a recent analysis provides informed insight into metal employment. An example is shown in Fig. 3, for the final uses of vanadium, tin, samarium, and rhenium circa 2020, with their alloy fractions varying between 70% and 97%. Similar information for all metals currently used in alloys or complexes is given in the Source Data file.

**Fig. 4:**
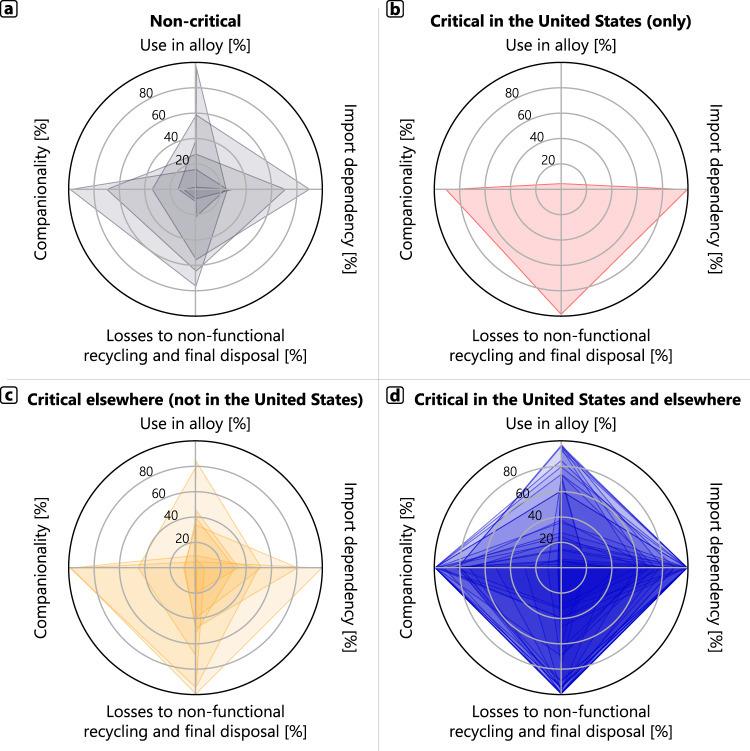
Radar charts for four criticality risk variables for 69 elements: use in alloy forms, U.S. import dependency, losses to nonfunctional recycling and final disposal, and companionality. **a** Elements not designated as critical by any country (six elements), (**b**) elements designated as critical by the United States only (one element), (**c**) elements designated as critical by other countries but not the United States (nine elements), and (**d**) elements designated as critical by the United States and one or more other countries (53 elements).

Four risk factors for the commonly used elements that are the focus of the present study can be displayed on a series of “radar plots”, as shown in Fig. 4 for four groups: noncritical elements that typically see significant use in alloy forms, elements designated critical only by the United States, elements designated critical only by countries other than the United States, and elements designated critical by both the United States and other countries. When the parameters for each element are overlaid with those of the other elements in that group, the intensity of the color indicates the commonality of the risk-parameter assessments among the elements in that group. (Numerical values for the variables’ alloy share, companionality, losses to nonfunctional recycling, and final disposal, and U.S. import dependency is given in Supplementary Table 4 of the Supplementary Information).

The combined radar plots for noncritical elements appear in Fig. 4a. A key feature of the diagram is that the functional end-of-life recycling rate (i.e., the remainder of the losses to nonfunctional recycling and final disposal) is relatively high (>60%), and there is essentially low companionality—that is, these elements are mostly mined for themselves. Other risk areas vary in intensity, but generally indicate relatively high use in alloys forms (≥50%) and high U.S. import dependence (60–80%).

Elements designated critical by the United States but not by other countries are plotted in Fig. 4b. This happens to be just one element: arsenic. The results show that arsenic is not used in alloys, mined entirely as a companion (mostly of zinc), its use in the United States depends fully on imports, and it is only modestly recycled at end-of-life (15%).

Elements designated critical by Australia, Canada, the European Union, and/or Japan but not the United States are plotted in Fig. 4c. Use in alloy forms is again relatively high (≥40%). Companionality is in the 40–60% range—much higher than for the noncritical elements. U.S. import dependence is similar. Functional end-of-life recycling is relatively low.

Finally, elements designated critical by the United States and other countries are shown in Fig. 4d. There is almost no functional end-of-life recycling for many of these elements. Companionality and U.S. import dependence are very high (80–100%). Use in alloy forms is slightly lower (~30–50%) than in the other groupings, probably because these elements often have specialized nonalloy uses in applications such as electronics or chemicals (cf. Supplementary Data file for details).

By employing the statistics used to construct Fig. 4, the elements can be examined individually, which we do in Supplementary Table 4. The results demonstrate that elements designated as critical by either or several governmental agencies tend to have low-to-very-low functional recycling, moderate-to-high import dependence, high companionality, and relatively high fractional use in alloy forms.

**Fig. 5:**
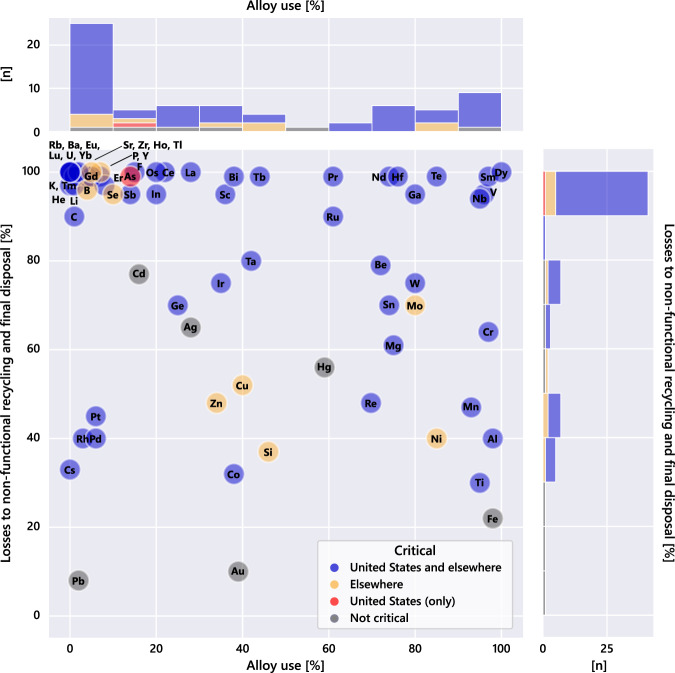
Losses to nonfunctional recycling and final disposal (i.e., 1—end-of-life recycling rate) as a function of alloy use for the elements in this analysis. The histogram plots on the top and right side count the number of elements in brackets of 10%.

An alternative analysis of this information is shown in Fig. 5, where alloy use is plotted against the degree of losses to nonfunctional end-of-life recycling rate and final disposal for the elements. This approach singles out six elements in the upper-right quadrant (>80% alloy use and nonrecovery): dysprosium, samarium, vanadium, niobium, tellurium, and gallium. Another five elements with high alloy use (>70%) and low functional recycling (<30%) are hafnium, neodymium, tin, tungsten, and beryllium, and molybdenum forms a second grouping of interest.

## Discussion

If materials contain metals deemed to be critical, a frequent recommendation (e.g.,^29,30^) is that those materials be targeted for functional end-of-life recycling (e.g., recycling that preserves the properties of the contained metals rather than merging them into larger flows in which the properties are lost or even degraded). However, without end-of-life concerns being considered by materials scientists, it seems likely that many modern alloys, especially high-complexity alloys, will see only a single use and then be lost or nonfunctionally recycled.

From the above perspective, it is interesting to reflect upon the reuse potential of elements in different regions of Fig. 5. Niobium and vanadium, for example, appear in the upper-right quadrant, characterized by very high alloy-use fractions and very low functional recycling rates. Almost all of their alloy uses are as minor fractions of a variety of iron alloys, most of which are inadvertently merged with carbon steels during recycling and their functionality is thereby lost. Rhenium is characterized by a high alloy fraction (70%) and a moderate-to-high functional recycling rate. Rhenium’s alloy use is entirely in superalloys—high in value and efficiently tracked and recovered^31^. Almost all of chromium (97%) is used in alloy form in stainless and alloy steels, yet the end-of-life recycling rate is only 36% since chromium recovery is limited to austenitic und selected ferritic stainless steels^32^. An additional consideration is that nonfunctional chromium recycling results in degraded iron-alloy performance^33,34^. We note that many elements in the upper-left quadrant of Fig. 5 are also poorly recycled at present, however, their nonalloy uses generally make them better targets for recycling and reuse.

The implication of all this is that the use of critical metals in low concentrations in alloys unlikely to be routinely recycled should be avoided if possible. If not, provision should be made for better identification and more efficient recycling so that materials designated as critical can have increased potential for more than a single functional use.

The designation of critical materials in order to promote “economic growth, the quality of life, national defense, and the functioning of modern society”^10^ and to “ensure jobs and competitiveness”^35^ is intended to benefit modern technology and product design, manufacture, use, and continual availability. Nonetheless, the issue of raw materials’ supply, in sufficient quantities now and in the future and at affordable prices, has been slow to resonate with the materials science community^36–38^ despite numerous examples of supply inadequacies over the past few decades. (Examples of supply shortages include cobalt^39^, rhenium^25^, and the rare earths^40^). Recently, however, materials’ availability has begun to be of more interest to materials scientists. In a recent insightful publication, Raabe et al.^41^ advocate building recyclability into the design of industrial materials, in part by using materials from potentially useful material compositions. We would add another crucial observation: that several technologically attractive metals are themselves critical, and their use should be avoided or minimized. If this latter recipe is followed, it is possible to imagine a more recyclable and more sustainable world in the future.

On a related topic, Fig. 1 demonstrated earlier in this paper that many elements in the periodic table have been labeled “critical”. In such a situation, the word “critical” begins to lose its meaning, a situation that brings to mind a line from the operetta “The Gondoliers”^42^: “When everyone is somebody, then no one’s anybody”. As shown herein, the realization that alloying tends to complicate the materials’ sustainability problem begins to provide useful perspective, one that has enabled a small number of metals to be singled out for particular attention. As technology evolves, traditional approaches to criticality assessment will likely evolve as well, but the discussion here can perhaps provide useful guidance in adding nuance to the designation of such a large fraction of metals and other materials as “critical” by consideration of the degree of alloying and the prospects for increases in functional recycling.

## Methods

### Fractional alloy use

To understand to what extent elements are used in alloy as opposed to nonalloy form the major end uses per metal have to be identified, and for each end use the share of alloy use has to be estimated. The alloy use share per element is then calculated as per Eq. 1:

1 $${{{{{{{{\rm{alloy}}}}}}\_{{{{{\rm{share}}}}}}}}}_{i}=\frac{\mathop{\sum }\limits_{j=1}^{n}{{{{{{{{\rm{end}}}}}}\_{{{{{\rm{use}}}}}}}}}_{i,j}}{{{{{{{{{\rm{end}}}}}}\_{{{{{\rm{use}}}}}}}}}_{{{{{{{\mathrm{total}}}}}}},{i}}}$$

where end_use_*i,j*_ indicates the end use in alloy share of the ith element of the periodic table in the jth end use, and end_use_total*,i*_ indicates the sum of all end uses of the ith element used in a year for a specific element in both alloy and nonalloy forms.

Information on the fractional use in alloy form, summarized in Supplementary Table 3 and detailed in the Source Data file, draws on governmental agency reports, industrial organization information, and scholarly publications (all cited in the Supplementary Information), with the fractional uses designated by us as “use in alloy form” or “use in nonalloy form”. These reports inform the fractional use of elements into specific uses (e.g., in 2016, lithium was used in batteries (43%), ceramic and glass products (28%), lubricant and grease (7%), synthetic rubber (5%), aluminum production (4%), air conditioners (3%), pharmaceuticals (2%), defense applications (~0%), and other uses (8%)^43^). Because specific data on material use do not explicitly state the form (alloy or nonalloy) of a use, and because forms of use evolve over time, our determinations are inherently the products of today’s expert judgment and should not be viewed as valid in perpetuity as product design is constantly changing. Nonetheless, we regard the results that we derive as valid representations of the state of play of contemporary technology, and quite adequate to support the principal conclusions of this work.

In general, we account all uses in electronics, transportation, structural components, industrial machinery, and soldering as alloy uses. Conversely, uses as catalysts, solutions, lubricants, glass, rubber, and others are categorized as nonalloy uses. A detailed report on the enduses of the 69 elements included in this study, as well as which of these end uses we considered as alloy use, is available in the Source Data file.

### End-of-life recycling rate

A metal is deemed more critical when recycling is low and the metal supply relies predominantly on primary sources. A low end-of-life recycling rate is thus of greater concern, which is why the risk of little recycling is expressed as the inverse of the end-of-life recycling rate, i.e., as losses to nonfunctional recycling (also termed downcycling) and final disposal (e.g., in landfills). The starting point to collect these data was a report of the UNEP’s International Resource Panel^44^. The report covers 60 metals and refers to the period 2005–2010. We supplemented the results of that report to reflect the status of the metal industries 10 years later and to extend the scope to 69 elements. New elements covered are helium, carbon (graphite), fluoride, silicon metal, phosphorus, potassium, rubidium, cesium, and uranium. This was accomplished by literature review^45–47^, oral or online interviews with government and industry specialists and, if necessary, by our judgments concerning data quality.

We calculated the end-of-life recycling rate (EOL-RR) using Eq. 2:

2 $${{{{{{{{\rm{EOL}}}}}}\_{{{{{\rm{RR}}}}}}}}}_{i}=\mathop{\sum }\limits_{j=1}^{n}{{{{{{{{\rm{end}}}}}}\_{{{{{\rm{use}}}}}}}}}_{i,j}\cdot {{{{{{{{\rm{EOL}}}}}}\_{{{{{\rm{RR}}}}}}}}}_{i,j}$$

where EOL_RR_*i*_ indicates the end-of-life recycling rate of the ith element of the periodic table, end_use_*i,j*_ denotes the percentage of the jth end use of the ith element, and EOL_RR_*i,j*_ indicates the end-of-life recycling rate of the jth end use of the ith element. Figures 4 and 5 plot the inverse of the EOL-RR, i.e., the percentage of losses to nonfunctional end-of-life recycling and final disposal, which are calculated using Eq. 3

3 $${{{{{{\rm{losses}}}}}}}_{i}=1-{{{{{{\rm{EOL}}}}}}}\_{{{{{\rm{RR}}}}}}_{i}$$

where losses_*i*_ designates the losses of the ith element of the periodic table to nonfunctional recycling and final disposal, and EOL_RR_*i*_ indicates the end-of-life recycling rate of the ith element.

### Companionality

Companionality is defined as “the degree to which a metal is obtained largely or entirely as a by-product of one or more host metals from geologic ores”^48^. In this study, we use data on the degree of companionality of metals as reported by Nassar et al.^48^, adding data for those elements that were not included in the original study (e.g., helium, fluorine, and silicon).

### Import dependence

Import dependence is calculated as the fraction of minerals used in industrial processes in the United States that comes from imports. Thus, an import dependence of 100% for a certain element indicates that the country completely relies on imports of chemicals and concentrates and that no active extractive plants exist inside its territories. Data on import dependence are routinely reported by the United States Geological Survey in its annual Minerals Yearbook^49^. In this study we use 2020 data.

## Supplementary information

Supplementary Information

## Data Availability

The data generated in this study are provided in the Source Data file. Source data are provided with this paper.

## Source data

Source Data
