# Supplementary material for: Alloy information helps prioritize material criticality lists
Source: Nat Commun. 2022 Jan 10;13:150. doi: 10.1038/s41467-021-27829-w (PMC8748655; doi:10.1038/s41467-021-27829-w)
Supplement: Supplementary file 1 — Supplementary Information [file 41467_2021_27829_MOESM1_ESM.docx]

Alloy Information Helps Prioritize
Material Criticality Lists

T. E. Graedel^*^, Barbara K. Reck, and Alessio Miatto

Center for Industrial Ecology, School of the Environment,
Yale University, New Haven, Connecticut 06511

**^*^** Corresponding Author: thomas.graedel@yale.edu

Supplementary Information

**Table of Contents**

[Supplementary notes 2](#_Toc87351897)

[Elements designated critical by various government entities 2](#_Toc87351898)

[Characteristics of elements deemed “Not Critical” 9](#_Toc87351899)

[Supplementary methods 10](#_Toc87351900)

[Fractional Use of Critical Elements in Alloy Forms 10](#_Toc87351901)

[Assessing Enhanced Concern for Critical Elements 15](#_Toc87351902)

[Supplementary References 20](#_Toc87351903)

# Supplementary notes

## Elements designated critical by various government entities

Supplementary Table 1 reports a list of all the elements of the periodic table and which of these elements are designated as critical by government entities ^1, 2, 3, 4, 5^. Supplementary Figures 1-5 display the same information on the periodic table. Supplementary Figure 6 reports a periodic table showing the frequency of criticality designation for each element.

Supplementary Table 1 – Elements designated critical by the governments of Australia ^1^, Canada ^3^, the European Union ^2^, Japan ^4^, and the United States ^5^.

| Atomic number | Symbol | Element | Critical in Australia | Critical in Canada | Critical in the European Union | Critical in Japan | Critical in the United States |
| --- | --- | --- | --- | --- | --- | --- | --- |
| 1 | H | Hydrogen |  |  |  |  |  |
| 2 | He | Helium | x | x |  |  | x |
| 3 | Li | Lithium | x | x | x | x | x |
| 4 | Be | Beryllium | x |  | x | x | x |
| 5 | B | Boron |  |  | x | x |  |
| 6 | C | Carbon | x | x | x | x | x |
| 7 | N | Nitrogen |  |  |  |  |  |
| 8 | O | Oxygen |  |  |  |  |  |
| 9 | F | Fluorine |  | x | x | x | x |
| 10 | Ne | Neon |  |  |  |  |  |
| 11 | Na | Sodium |  |  |  |  |  |
| 12 | Mg | Magnesium | x | x | x | x | x |
| 13 | Al | Aluminium |  | x | x |  | x |
| 14 | Si | Silicon |  |  | x | x |  |
| 15 | P | Phosphorus |  |  | x |  |  |
| 16 | S | Sulfur |  |  |  |  |  |
| 17 | Cl | Chlorine |  |  |  |  |  |
| 18 | Ar | Argon |  |  |  |  |  |
| 19 | K | Potassium |  | x |  |  | x |
| 20 | Ca | Calcium |  |  |  |  |  |
| 21 | Sc | Scandium | x | x | x | x | x |
| 22 | Ti | Titanium | x | x | x | x | x |
| 23 | V | Vanadium | x | x | x | x | x |
| 24 | Cr | Chromium | x | x |  | x | x |
| 25 | Mn | Manganese | x | x |  | x | x |
| 26 | Fe | Iron |  |  |  |  |  |
| 27 | Co | Cobalt | x | x | x | x | x |
| 28 | Ni | Nickel |  | x |  | x |  |
| 29 | Cu | Copper |  | x |  |  |  |
| 30 | Zn | Zinc |  | x |  |  |  |
| 31 | Ga | Gallium | x | x | x | x | x |
| 32 | Ge | Germanium | x | x | x | x | x |
| 33 | As | Arsenic |  |  |  |  | x |
| 34 | Se | Selenium |  |  |  | x |  |
| 35 | Br | Bromine |  |  |  |  |  |
| 36 | Kr | Krypton |  |  |  |  |  |
| 37 | Rb | Rubidium |  |  |  | x | x |
| 38 | Sr | Strontium |  |  | x | x | x |
| 39 | Y | Yttrium | x | x | x | x | x |
| 40 | Zr | Zirconium | x |  |  | x | x |
| 41 | Nb | Niobium | x | x | x | x | x |
| 42 | Mo | Molybdenum |  | x |  | x |  |
| 43 | Tc | Technetium |  |  |  |  |  |
| 44 | Ru | Ruthenium | x | x | x | x | x |
| 45 | Rh | Rhodium | x | x | x | x | x |
| 46 | Pd | Palladium | x | x | x | x | x |
| 47 | Ag | Silver |  |  |  |  |  |
| 48 | Cd | Cadmium |  |  |  |  |  |
| 49 | In | Indium | x | x | x | x | x |
| 50 | Sn | Tin |  | x |  |  | x |
| 51 | Sb | Antimony | x | x | x | x | x |
| 52 | Te | Tellurium |  | x |  | x | x |
| 53 | I | Iodine |  |  |  |  |  |
| 54 | Xe | Xenon |  |  |  |  |  |
| 55 | Cs | Caesium |  | x |  | x | x |
| 56 | Ba | Barium |  |  | x | x | x |
| 57 | La | Lanthanum | x | x | x | x | x |
| 58 | Ce | Cerium | x | x | x | x | x |
| 59 | Pr | Praseodymium | x | x | x | x | x |
| 60 | Nd | Neodymium | x | x | x | x | x |
| 61 | Pm | Promethium |  |  |  |  |  |
| 62 | Sm | Samarium | x | x | x | x | x |
| 63 | Eu | Europium | x | x | x | x | x |
| 64 | Gd | Gadolinium | x | x | x | x | x |
| 65 | Tb | Terbium | x | x | x | x | x |
| 66 | Dy | Dysprosium | x | x | x | x | x |
| 67 | Ho | Holmium | x | x | x | x | x |
| 68 | Er | Erbium | x | x | x | x | x |
| 69 | Tm | Thulium | x | x | x | x | x |
| 70 | Yb | Ytterbium | x | x | x | x | x |
| 71 | Lu | Lutetium | x | x | x | x | x |
| 72 | Hf | Hafnium | x |  | x | x | x |
| 73 | Ta | Tantalum | x | x | x | x | x |
| 74 | W | Tungsten | x | x | x | x | x |
| 75 | Re | Rhenium | x |  |  | x | x |
| 76 | Os | Osmium | x | x | x | x | x |
| 77 | Ir | Iridium | x | x | x | x | x |
| 78 | Pt | Platinum | x | x | x | x | x |
| 79 | Au | Gold |  |  |  |  |  |
| 80 | Hg | Mercury |  |  |  |  |  |
| 81 | Tl | Thallium |  |  |  | x |  |
| 82 | Pb | Lead |  |  |  |  |  |
| 83 | Bi | Bismuth | x | x | x | x | x |
| 84 | Po | Polonium |  |  |  |  |  |
| 85 | At | Astatine |  |  |  |  |  |
| 86 | Rn | Radon |  |  |  |  |  |
| 87 | Fr | Francium |  |  |  |  |  |
| 88 | Ra | Radium |  |  |  |  |  |
| 89 | Ac | Actinium |  |  |  |  |  |
| 90 | Th | Thorium |  |  |  |  |  |
| 91 | Pa | Protactinium |  |  |  |  |  |
| 92 | U | Uranium |  | x |  |  | x |


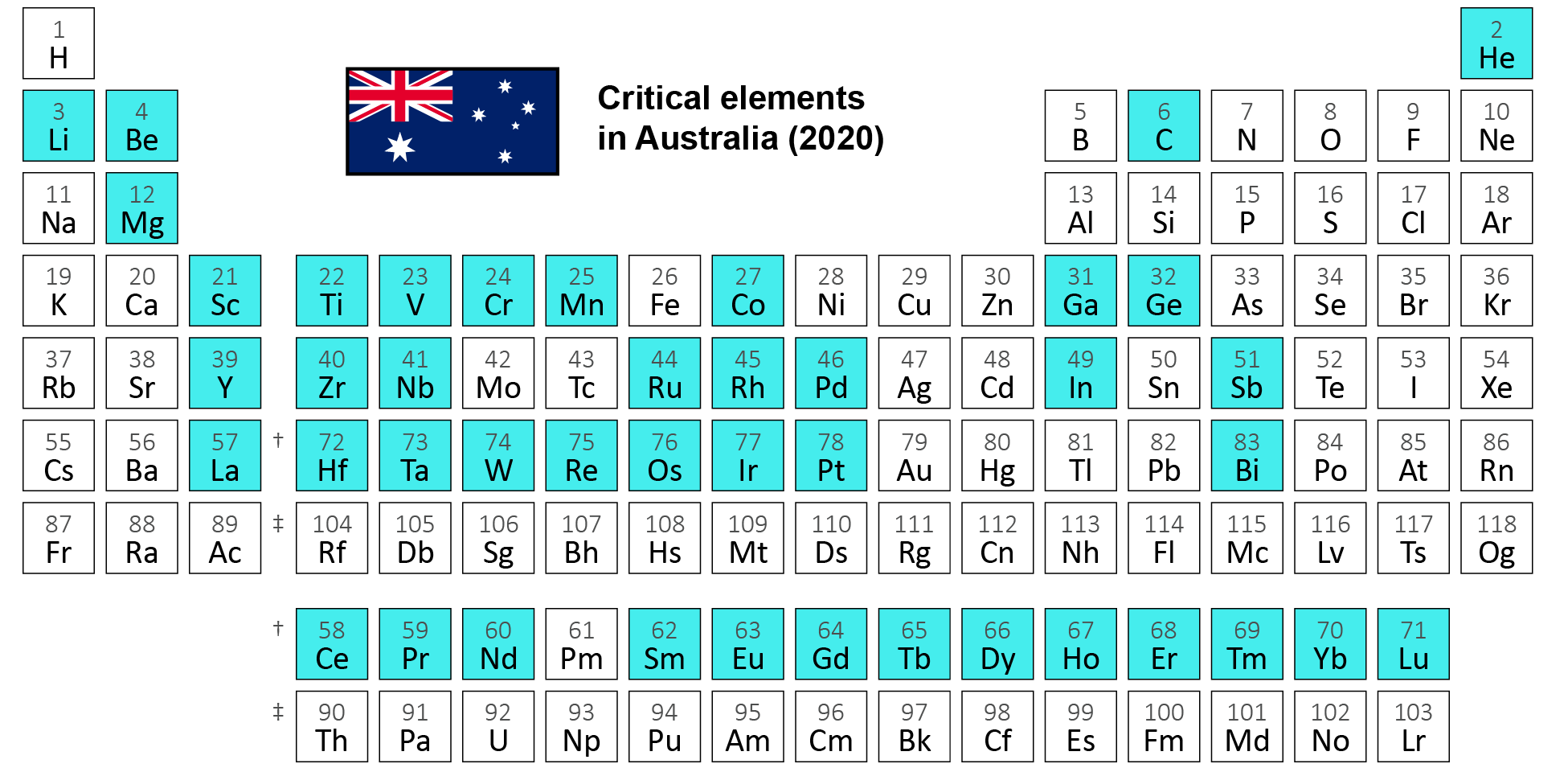


Supplementary Figure 1 – Periodic table of elements designated critical by the Australian Government (list of 2020).


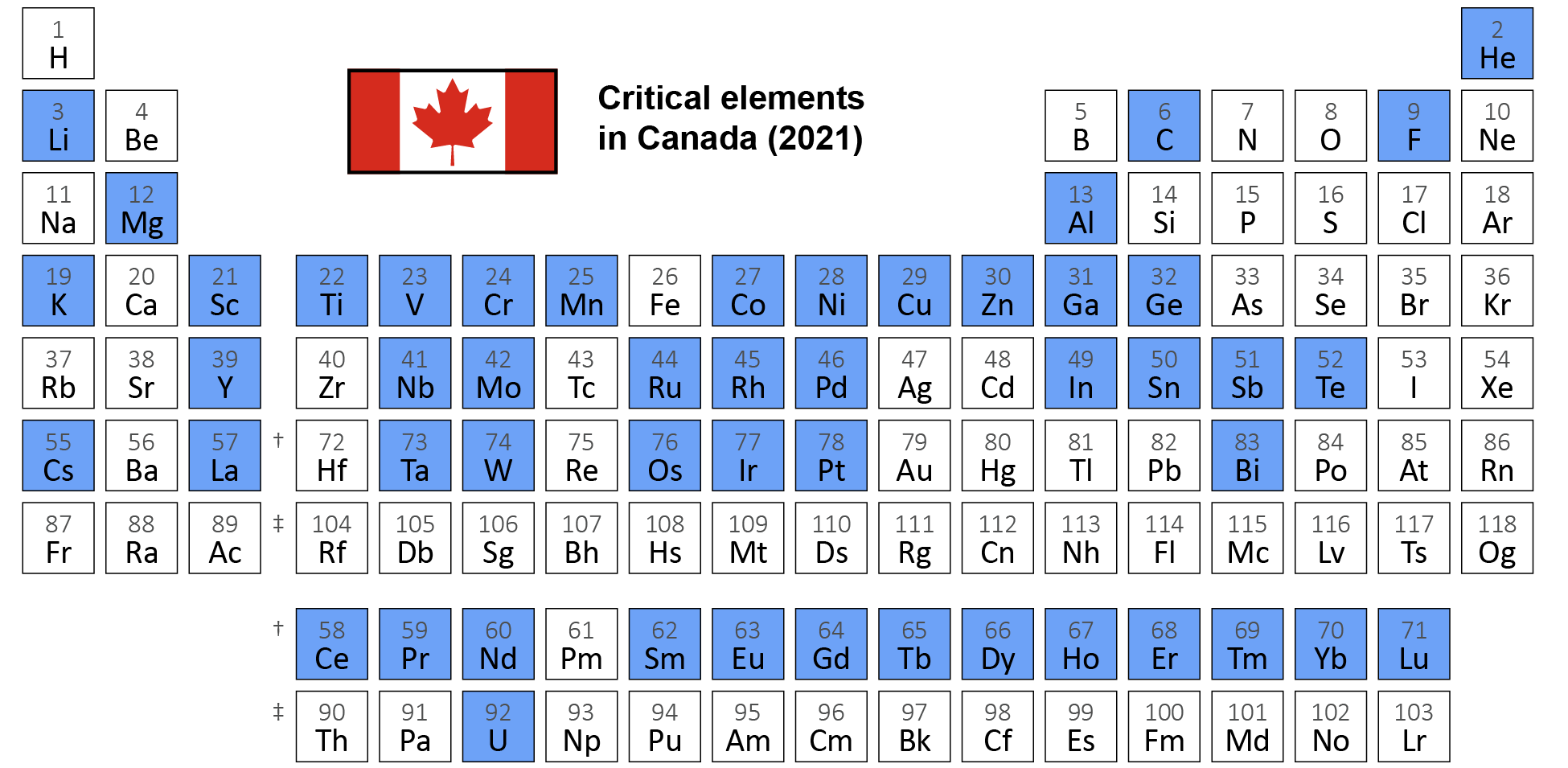


Supplementary Figure 2 – Periodic table of elements designated critical by the Canadian Government (list of 2021).


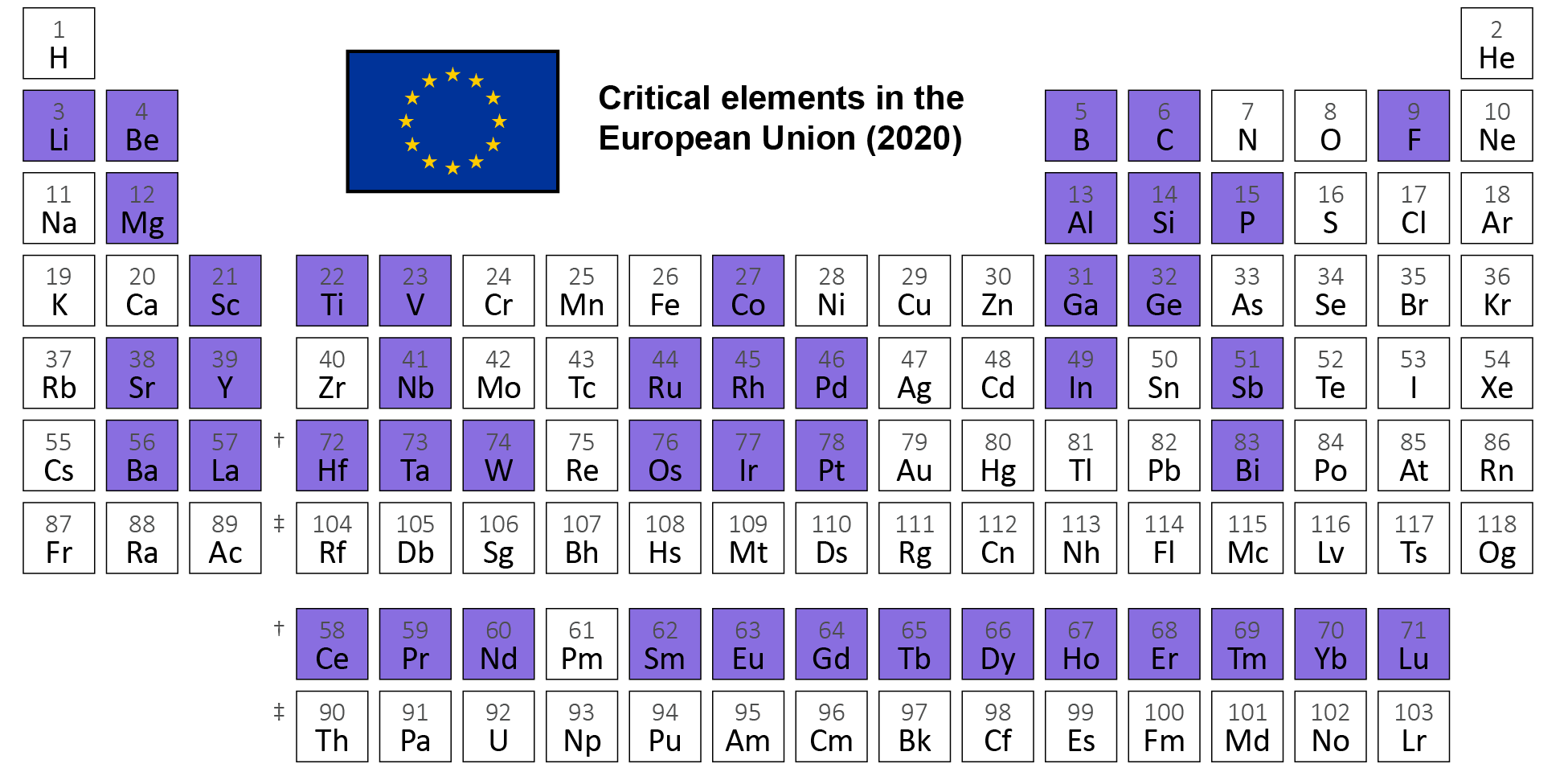


Supplementary Figure 3 – Periodic table of elements designated critical by the European Union (list of 2020).


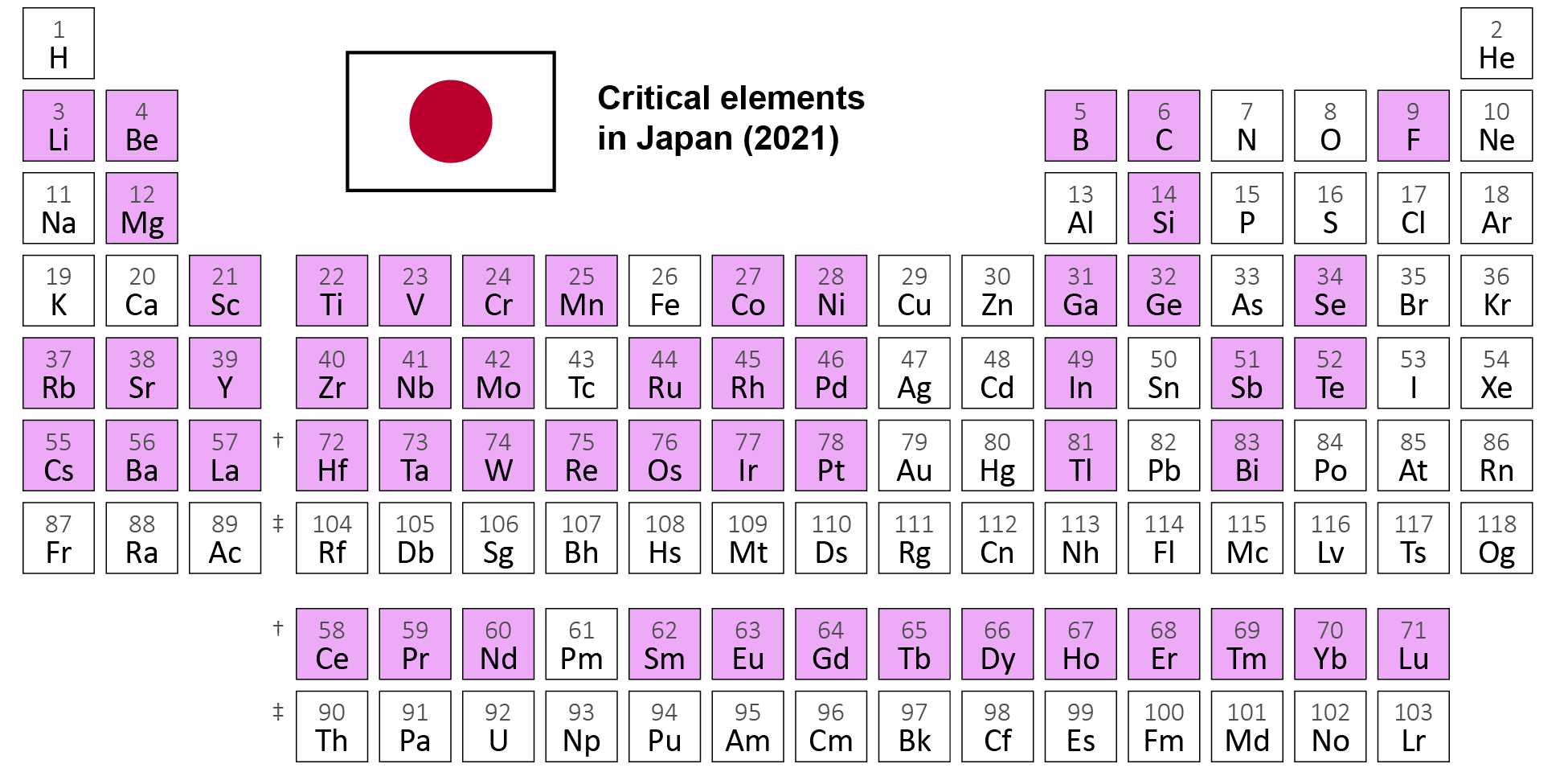


Supplementary Figure 4 – Periodic table of elements designated critical by the Japanese Ministry of Economy, Trade and Industry (list of 2021).


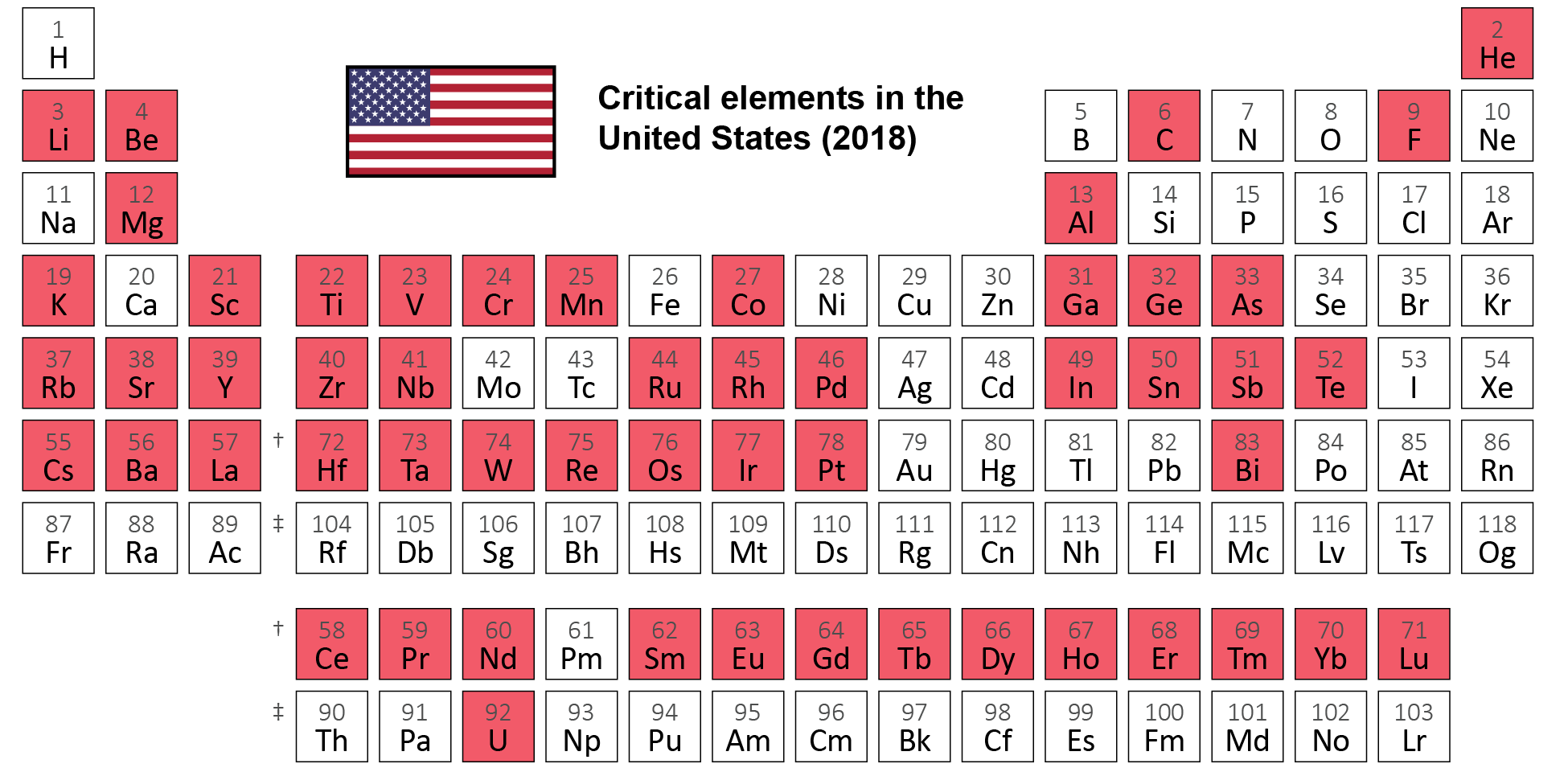


Supplementary Figure 5 – Periodic table of elements designated critical by the U.S. Department of the Interior (list of 2018).


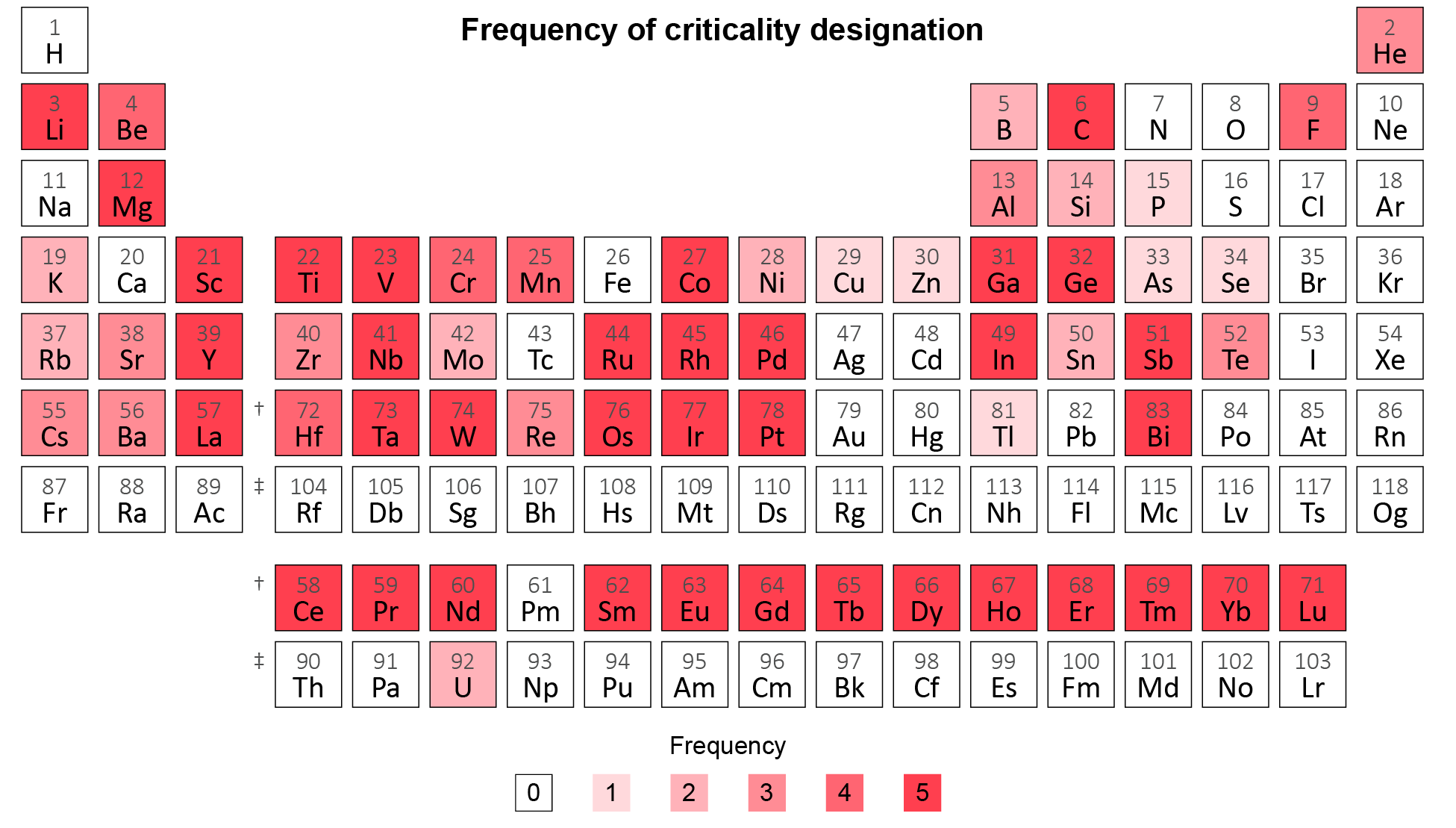


Supplementary Figure 6 – Periodic table showing the frequency of criticality designation.

## Characteristics of elements deemed “Not Critical”

As noted in the main manuscript, it is interesting to ask which metals or metalloids (the alloy formers) *do not* appear on lists of critical materials. It turns out that from the designations of Australia, Canada, European Union, Japan, and the United States, there are only six such metals: iron, silver, cadmium, gold, mercury, and lead. The lack of concern for these elements relates to the fact that two have relatively high crustal abundances, while others are marked by attributes of high toxicity, and companionality (the degree to which a metal is obtained largely or entirely as a by-product of one or more host metals in geological ores). Even though cadmium is very scarce and available only as a companion, its high toxicity is sufficient to qualify it as not critical (Supplementary Table 2).

Supplementary Table 2 – Relevant characteristics for six elements deemed NOT critical by various governmental agencies.

| Characteristic | Fe | Ag | Cd | Au | Hg | Pb |
| --- | --- | --- | --- | --- | --- | --- |
| Abundant ^*^ | x | x |  |  |  |  |
| Highly toxic ^@^ |  |  | x |  | x | x |
| Companion metal ^+^ |  | x | x |  |  |  |
| Very scarce ^#^ |  |  | x |  |  |  |
| Notes  ^*^ One of the ten metals of highest crustal abundance ^6^; the others are Mg, Al, Ti, V, Cr, Zn, Zr, and Ce.  ^@^ Emsley ^7^  ^+^ Defined as “companion metal” ^8^  ^#^ One of the ten metals of lowest crustal abundance ^6^; the others are Ru, Pd, In, Tm, Lu, Re, Os, Pt, and Bi. | | | | | | |

# Supplementary methods

## Fractional Use of Critical Elements in Alloy Forms

In Supplementary Table 3 we report the percentage of the alloy share of the 69 elements we included in our analysis and the source from which the alloy share was taken. A detailed breakdown of all the end-uses is reported in the Source Data file in the tab “End Uses”.

Supplementary Table 3 – Percentage of alloy use for 69 elements of the periodic table. The highlights in alternating blue/green denote the average percentage of alloy share.

| **Atomic number** | **Element** | **Symbol** | **Alloy share** | **Scope** | **Reference Year** | **Source** |
| --- | --- | --- | --- | --- | --- | --- |
| **2** | **Helium** | **He** | 0% | U.S. | 2018 | U.S. Geological Survey ^9^ |
|  |  |  | **0%** | **U.S.** | **2018** | **USGS 2018 ^9^** |
| **3** | **Lithium** | **Li** | 1% | U.S. | 2016 | Miatto et al. ^10^ |
|  |  |  | 1% | Global | 2017 | Sun et al. ^11^ |
|  |  |  | **1%** | **Global** | **2020** | **USGS 2021 ^12^** |
| **4** | **Beryllium** | **Be** | 74% | E.U. | 2017 | Best estimated based on European Commission ^13^ |
|  |  |  | 71% | U.S. | 2019 | Best estimate*,* based on U.S. Geological Survey ^14^ |
|  |  |  | **72%** | **Global** |  | **Best estimate*,* based on EU and USGS** |
| **5** | **Boron** | **B** | 4% | E.U. | 2010-2014 | European Commission ^13^ |
|  |  |  | 4% | U.S. | 2020 | U.S. Geological Survey ^12^ |
|  |  |  | **4%** | **Global** |  | **Best estimate*,* based on EU 2017 and USGS 2021** |
| **6** | **Carbon (graphite)** | **C** | 0% | U.S. | 2017 | U.S. Geological Survey ^15^ |
|  |  |  | 0% | Global | 2019 | Jara et al. ^16^ |
|  |  |  | **1%** | **Global** |  | **Best estimate** |
| **9** | **Fluorine** | **F** | 15% | Global | 2017 | U.S. Geological Survey ^15^ |
|  |  |  | **15%** | **Global** |  | **USGS Minerals Yearbook 2017 ^15^** |
| **12** | **Magnesium metal** | **Mg** | 75% | E.U. | 2010-2014 | Best estimate based on European Commission ^13^ |
|  |  |  | 82% | U.S. | 2020 | Best estimated based on U.S. Geological Survey ^12^ |
|  |  |  | 72% | Global | 2012 | Roskill Information Services ^17^ |
|  |  |  | **75%** | **Global** |  | ***Best estimate,* based on Roskill 2013 ^17^** |
| **13** | **Aluminium** | **Al** | 100% | E.U. | ca. 2015 | European Commission ^13^ |
|  |  |  | 97% | U.S. | 2020 | U.S. Geological Survey ^12^ |
|  |  |  | **98%** | **Global** |  | ***Best estimate,* based on USGS MCS 2021 ^12^** |
| **14** | **Silicon metal** | **Si** | 46% | E.U. | 2010-2014 | Best estimate based on European Commission ^13^ |
|  |  |  | **46%** | **Global** |  | ***Best estimate,* based on EU 2017 ^13^** |
| **15** | **Phosphorus** | **P** | 10% | Global | 2010-2014 | Best estimate based on European Commission ^13^ |
|  |  |  | **7%** | Global |  | *Best Estimate,* based on European Commission ^13^ |
| **19** | **Potassium** | **K** | 0% | U.S. | 2020 | U.S. Geological Survey ^12^ |
|  |  |  | **1%** | U.S. |  | **Best estimate** |
| **21** | **Scandium** | **Sc** | 9% | Global | 2010-2014 | Best estimate based on European Commission ^13^ |
|  |  |  | **36%** | Global | 2019 | Mordor Intelligence ^18^ |
| **22** | **Titanium** | **Ti** | 21% | Global | 2010-2014 | European Commission ^13^ |
|  |  |  | 95% | U.S. | 2017 | USGS 2017 Minerals Yearbook. Titanium ^19^ |
|  |  |  | **95%** | Global |  | ***Best estimate,* based on USGS 2017 MYB ^19^** |
| **23** | **Vanadium** | **V** | **96%** | Global | 2010-2014 | European Commission ^13^ |
| **24** | **Chromium** | **Cr** | 96% | E.U. | 2010-2014 | European Commission ^13^ |
|  |  |  | 98% | U.S. | 2019 | USGS Mineral Commodity Summaries 2020 ^14^ |
|  |  |  | **97%** | Global |  | ***Best estimate,* based on USGS MCS 2020 and EC 2017** |
| **25** | **Manganese** | **Mn** | **93%** | Global | 2014 | European Commission 2017 ^13^ |
| **26** | **Iron** | **Fe** | 98% | U.S. | 2020 | USGS Mineral Commodity Summaries 2021 ^12^ |
|  |  |  | **98%** | Global |  | ***Best estimate,* based on USGS MCS 2021** |
| **27** | **Cobalt** | **Co** | **38%** | Global | 2015 | European Commission 2017 ^13^ |
| **28** | **Nickel** | **Ni** | 64% | Global | 2014 | European Commission 2017 ^13^ |
|  |  |  | 86% | Global | 2010 | Reck and Rotter, 2012 ^20^ |
|  |  |  | **85%** | Global |  | ***Best estimate,* based on Reck and Rotter and European Commission 2017** |
| **29** | **Copper** | **Cu** | n/a | Global | 2010-2014 | European Commission 2017 ^13^ |
|  |  |  | 54% | Global | 2009 | Best estimate, based on Graedel et al. (2015) ^21^ |
|  |  |  | n/a | U.S. | 2018 | Copper Development Association (2020) ^22^ |
|  |  |  | **40%** | Global |  | ***Best estimate,* based on CDA (2020)** |
| **30** | **Zinc** | **Zn** | **34%** | Global | 2010-2014 | European Commission 2017 ^13^ |
| **31** | **Gallium** | **Ga** | 75% | Global | 2014 | European Commission 2017 ^13^ |
|  |  |  | 80% | U.S. | 2020 | USGS Mineral Commodity Summaries 2021 ^12^ |
|  |  |  | 93% | Global | 2011 | Licht et al. (2015) ^23^ |
|  |  |  | **80%** | Global |  | ***Best estimate,* based on Licht et al. (2015) and EC 2017** |
| **32** | **Germanium** | **Ge** | 13% | E.U. | 2012 | European Commission 2017 ^13^ |
|  |  |  | 12% | Global | 2002 | Moskalyk (2004) ^24^ |
|  |  |  | **25%** | Global | 2011 | **Licht et al. (2015) ^23^** |
| **33** | **Arsenic** | **As** | **14%** | Global | 2015 | Graedel et al. (2015) ^21^ |
| **34** | **Selenium** | **Se** | **10%** | Global | 2009-2016 | European Commission 2017 ^13^ |
| **37** | **Rubidium** | **Rb** | **0%** | U.S. | 2020 | USGS Mineral Commodity Summaries 2021 ^12^ |
| **38** | **Strontium** | **Sr** | 3% | U.S. | 2019 | USGS Mineral Commodity Summaries 2020 ^14^ |
|  |  |  | 5% | U.S. | 2020 | USGS Mineral Commodity Summaries 2021 ^12^ |
|  |  |  | **5%** | Global |  | ***Best estimate,* based on USGS MCS 2021** |
| **39** | **Yttrium** | **Y** | 7% | Global | 2010-2014 | European Commission 2017 ^13^ |
|  |  |  | 1% | Global | 2015 | Guyonett et al. (2015) ^25^ |
|  |  |  | **7%** | Global |  | ***Best estimate,* based on European Commission 2017** |
| **40** | **Zirconium** | **Zr** | n/a | U.S. | 2020 | USGS Mineral Commodity Summaries 2021 ^12^ |
|  |  |  | **5%** | Global |  | ***Best estimate,* based on USGS MCS 2021** |
| **41** | **Niobium** | **Nb** | 94% | Global | 2014 | European Commission 2017 ^13^ |
|  |  |  | 100% | U.S. | 2020 | USGS Mineral Commodity Summaries 2021 ^12^ |
|  |  |  | **95%** | Global |  | ***Best estimate,* based on USGS MCS 2021 and EC 2017** |
| **42** | **Molybdenum** | **Mo** | 79% | Global | 2010-2014 | European Commission 2017 ^13^ |
|  |  |  | **80%** | Global | 2018 | International Molybdenum Association (2020) ^26^ |
| **44** | **Ruthenium** | **Ru** | **61%** | Global | 2015 | European Commission 2017 ^13^ |
| **45** | **Rhodium** | **Rh** | n/a | Global | 2014 | European Commission 2017 ^13^ |
|  |  |  | **3%** | Global |  | ***Best estimate,* based on EU 2017** |
| **46** | **Palladium** | **Pd** | n/a | Global | 2015 | European Commission 2017 ^13^ |
|  |  |  | **6%** | Global |  | ***Best estimate,* based on EU 2017** |
| **47** | **Silver** | **Ag** | **28%** | Global | 2010-2014 | **European Commission 2017 ^13^** |
| **48** | **Cadmium** | **Cd** | **16%** | Global | 2019 | International Cadmium Association (2019) ^27^ |
| **49** | **Indium** | **In** | 17% | Global | 2012 | European Commission 2017 ^13^ |
|  |  |  | 16% | Global | 2011 | Licht et al. (2015) ^23^ |
|  |  |  | **20%** |  |  | ***Best estimate,* based on EU 2017 and Licht et al. (2015)** |
| **50** | **Tin** | **Sn** | 68% | Global | 2010-2014 | European Commission 2017 ^13^ |
|  |  |  | 55% | E.U. |  | European Commission 2017 ^13^ |
|  |  |  | 56% | U.S. | 2020 | USGS Mineral Commodity Summaries 2021 ^12^ |
|  |  |  | **74%** | Global | 2017 | International Tin Association (2018) ^28^ |
| **51** | **Antimony** | **Sb** | **14%** | Global | 2014 | European Commission 2017 ^13^ |
| **52** | **Tellurium** | **Te** | **85%** | Global | n/a | European Commission 2017 ^13^ |
| **55** | **Cesium** | **Cs** | 0% | U.S. | 2020 | USGS Mineral Commodity Summaries 2021 ^12^ |
|  |  |  | **0%** | U.S. |  | ***Best estimate,* based on MCS 2021** |
| **56** | **Barium** | **Ba** | 0% | E.U. | 2010-2014 | European Commission 2017 ^13^ |
|  |  |  | 0% | U.S. | 2020 | USGS Mineral Commodity Summaries 2021 ^12^ |
|  |  |  | **0%** | Global |  | ***Best estimate,* based on EU 2017 and USGS MCS 2021** |
| **57** | **Lanthanum** | **La** | 15% | E.U. | 2010-2014 | European Commission 2017 ^13^ |
|  |  |  | 0% | U.S. | 2020 | USGS Mineral Commodity Summaries 2021 ^12^ |
|  |  |  | 28% | Global | 2015 | Guyonett et al. (2015) ^25^ |
|  |  |  | **28%** | Global |  | ***Best estimate,* based on Guyonett et al. (2015)** |
| **58** | **Cerium** | **Ce** | 13% | E.U. | 2010-2014 | European Commission 2017 ^13^ |
|  |  |  | 22% | Global | 2015 | Guyonett et al. (2015) ^25^ |
|  |  |  | **22%** | Global |  | ***Best estimate,* based on Guyonett et al. (2015)** |
| **59** | **Praseo-dymium** | **Pr** | 35% | E.U. | 2010-2014 | European Commission 2017 ^13^ |
|  |  |  | 61% | Global | 2015 | Guyonett et al. (2015) ^25^ |
|  |  |  | **61%** | Global |  | ***Best estimate,* based on Guyonett et al. (2015)** |
| **60** | **Neodymium** | **Nd** | 49% | E.U. | 2010-2014 | European Commission 2017 ^13^ |
|  |  |  | 74% | Global | 2015 | Guyonett et al. (2015) ^25^ |
|  |  |  | **74%** | Global |  | ***Best estimate,* based on Guyonett et al. (2015)** |
| **62** | **Samarium** | **Sm** | 97% | E.U. | 2010-2014 | European Commission 2017 ^13^ |
|  |  |  | 0% | Global | 2015 | Guyonett et al. (2015) ^25^ |
|  |  |  | **97%** | Global |  | ***Best estimate,* based on EU 2017** |
| **63** | **Europium** | **Eu** | 0% | Global | 2015 | Guyonett et al. (2015) ^25^ |
|  |  |  | 0% | Global | 2020 | Grand View Research (2017) ^29^ |
|  |  |  | **0%** | Global |  | ***Best estimate,* based on Guyonett et al. (2015) and Gran View Research (2017)** |
| **64** | **Gadolinium** | **Gd** | 5% | U.S. | 2017 | USGS 2017 Minerals Yearbook (Rare Earths) ^30^ |
|  |  |  | 0% | E.U. | 2015 | Guyonett et al. (2015) ^25^ |
|  |  |  | **4%** | U.S. |  | **Best estimate, based on USGS 2017** |
| **65** | **Terbium** | **Tb** | **44%** | E.U. | 2015 | **Guyonett et al. (2015) ^25^** |
| **66** | **Dysprosium** | **Dy** | 100% | E.U. | 2010-2014 | European Commission 2017 ^13^ |
|  |  |  | 100% | Global | 2015 | Guyonett et al. (2015) ^25^ |
|  |  |  | **100%** | Global |  | ***Best estimate,* based on EU 2017 and Guyonett et al. 2015** |
| **67** | **Holmium** | **Ho** | **5%** | U.S. | 2017 | **USGS 2017 Minerals Yearbook (Rare Earths) ^30^** |
| **68** | **Erbium** | **Er** | **8%** | Global | 2020 | **Avalon Advanced Materials (2021)^31^** |
| **69** | **Thulium** | **Tm** | **1%** | Global | 2008 | **Thulium is a glass act (2008) ^32^** |
| **70** | **Ytterbium** | **Yb** | **2%** | Global | 2012 | **AZO Materials (2012) ^33^** |
| **71** | **Lutetium** | **Lu** | **0%** | Global |  | **AZO Materials (2012) ^34^** |
| **72** | **Hafnium** | **Hf** | **76%** | Global | 2010-2014 | **European Commission 2017 ^13^** |
| **73** | **Tantalum** | **Ta** | 39% | Global | 2010-2014 | European Commission 2017 ^13^ |
|  |  |  | 42% | Global | 2012 | Roskill (2013) ^35^ |
|  |  |  | **42%** | Global |  | ***Best estimate,* based on Roskill 2013** |
| **74** | **Tungsten** | **W** | 81% | E.U. | 2010-2014 | European Commission 2017 ^13^ |
|  |  |  | 80% | Europe | 2010 | MSP-REFRAM (2020) ^36^ |
|  |  |  | n/a | U.S. | 2020 | USGS Mineral Commodity Summaries 2021 ^12^ |
|  |  |  | **80%** | Global |  | ***Best estimate,* based on EU 2017 and MSP-REFRAM (2020)** |
| **75** | **Rhenium** | **Re** | 61% | Global | 2010-2014 | European Commission 2017 ^13^ |
|  |  |  | 78% | Global | 2011 | MSP-REFRAM (2020) ^37^ |
|  |  |  | **70%** | Global |  | ***Best estimate,* based on EU 2017 and MSP-REFRAM 2020** |
| **76** | **Osmium** | **Os** | n/a | Global | 2015 | Graedel et al. (2015) ^21^ |
|  |  |  | **20%** | Global |  | ***Best estimate*, based on** **Graedel et al. (2015)** |
| **77** | **Iridium** | **Ir** | 43% | Global | 2015 | European Commission 2017 ^13^ |
|  |  |  | 15% | Global | 2012 | Johnson Matthey (2013) Platinum 2013 (Johnson Matthey, Royston, United Kingdom). In ^21^ |
|  |  |  | 36% | Global | 2018 | *Estimate,* based on PGM Market Report (2020) ^38^ |
|  |  |  | 35% | Global | 2020 | *Estimate,* based on S&P Global (2021) ^39^ |
|  |  |  | **35%** | Global |  | ***Best estimate,* based on S&P Global (2021) and PGM Market Report (2020)** |
| **78** | **Platinum** | **Pt** | **6%** | Global | 2015 | **European Commission 2017 ^13^** |
| **79** | **Gold** | **Au** | 85% | Global | 2010-2014 | European Commission 2017 ^13^ |
|  |  |  | **39%** | Global | 2020 | **USGS Mineral Commodity Summaries 2021 ^12^** |
| **80** | **Mercury** | **Hg** | n/a | U.S. | 2020 | USGS Mineral Commodity Summaries 2021 ^12^ |
|  |  |  | **59%** |  |  | ***Best estimate,* based on USGS MCS 2021** |
| **81** | **Thallium** | **Tl** | n/a | U.S. | 2020 | USGS Mineral Commodity Summaries 2021 ^12^ |
|  |  |  | **5%** | Global |  | ***Best estimate,* based on USGS MCS 2021** |
| **82** | **Lead** | **Pb** | 2% | Global | 2012 | European Commission 2017 ^13^ |
|  |  |  | 5% | Global | 2020 | International Lead and Zinc Study Group (2021) ^40^ |
|  |  |  | **2%** | Global | 2019 | **NRCan (2021) ^41^** |
| **83** | **Bismuth** | **Bi** | **38%** | Global | 2014 | **European Commission 2017 ^13^** |
| **92** | **Uranium** | **U** | 0% | Global | 2021 | Royal Society of Chemistry (2021) ^42^ |
|  |  |  | **0%** | Global |  | ***Best estimate,* based on Royal Society of Chemistry** |

## Assessing Enhanced Concern for Critical Elements

As noted in the main publication, the criticality issue is not very well focused when nearly all possible elements or materials are so designated. We have presented in the present work, however, several metrics of concern that could be regarded as complementary and/or supportive to the various criticality methodologies. We here combine these metrics to derive a more selective group of elements about whose future we might regard with enhanced concern. In doing so we do not pretend that the results are rigorous, but rather that they may offer an improved perspective on the issue of materials criticality.

We here evaluated, element by element, the four metrics of Figure 3 in the main manuscript, displaying their numerical values. The results are shown in Supplementary Table 4. The values of companionality were taken from Nassar et al. (2015) ^8^; the recycling rates were sourced from the UNEP’s International Resource Panel (2011) ^43^ (herein we use the remainder of the percentage to focus on how much is not recycled); import dependencies for the United States were collected from various publications of the U.S. Geological Survey Mineral Commodity Summaries ^9, 12, 14, 15^; data for use in alloy form were retrieved from multiple sources, which are listed in section 2.1 of this document.

Supplementary Table 4 – Metal by metal statistics on the elements of Figure 3 of the main manuscript.

| **Atomic number** | **Element** | **Symbol** | **Alloy use [%]** | **Companionality [%]** | **Losses to downcycling and final disposal [%]** | **U.S. import dependency [%]** | **Critical where?** |
| --- | --- | --- | --- | --- | --- | --- | --- |
| 2 | Helium | He | 0 | 0 | 97 | 0 | United States and elsewhere |
| 3 | Lithium | Li | 1 | 52 | 97 | 60 | United States and elsewhere |
| 4 | Beryllium | Be | 72 | 11 | 79 | 20 | United States and elsewhere |
| 5 | Boron | B | 4 | 0.3 | 96 | 1 | Elsewhere |
| 6 | Carbon (graphite) | C | 1 | 0 | 90 | 100 | United States and elsewhere |
| 9 | Fluorine | F | 15 | 80 | 100 | 100 | United States and elsewhere |
| 12 | Magnesium | Mg | 75 | 3 | 61 | 20 | United States and elsewhere |
| 13 | Aluminium | Al | 98 | 1 | 40 | 81 | United States and elsewhere |
| 14 | Silicon metal | Si | 46 | 0 | 37 | 34 | Elsewhere |
| 15 | Phosphorus | P | 7 | 1 | 100 | 7 | Elsewhere |
| 19 | Potassium | K | 1 | 0 | 100 | 90 | United States and elsewhere |
| 21 | Scandium | Sc | 36 | 100 | 95 | 100 | United States and elsewhere |
| 22 | Titanium metal | Ti | 95 | 1 | 30 | 61 | United States and elsewhere |
| 23 | Vanadium | V | 96 | 82 | 95 | 100 | United States and elsewhere |
| 24 | Chromium | Cr | 97 | 2 | 64 | 69 | United States and elsewhere |
| 25 | Manganese | Mn | 93 | 3 | 47 | 100 | United States and elsewhere |
| 26 | Iron | Fe | 98 | 0.5 | 22 | 24 | Not critical |
| 27 | Cobalt | Co | 38 | 85 | 32 | 72 | United States and elsewhere |
| 28 | Nickel | Ni | 85 | 2 | 40 | 52 | Elsewhere |
| 29 | Copper | Cu | 40 | 9 | 52 | 31 | Elsewhere |
| 30 | Zinc | Zn | 34 | 10 | 48 | 81 | Elsewhere |
| 31 | Gallium | Ga | 80 | 100 | 95 | 100 | United States and elsewhere |
| 32 | Germanium | Ge | 25 | 100 | 70 | 65 | United States and elsewhere |
| 33 | Arsenic | As | 14 | 92 | 99 | 100 | United States (only) |
| 34 | Selenium | Se | 10 | 100 | 95 | 50 | Elsewhere |
| 37 | Rubidium | Rb | 0 | 100 | 100 | 100 | United States and elsewhere |
| 38 | Strontium | Sr | 5 | 0 | 99 | 100 | United States and elsewhere |
| 39 | Yttrium | Y | 7 | 29 | 99 | 100 | United States and elsewhere |
| 40 | Zirconium | Zr | 5 | 100 | 99 | 40 | United States and elsewhere |
| 41 | Niobium | Nb | 95 | 2 | 94 | 100 | United States and elsewhere |
| 42 | Molybdenum | Mo | 80 | 46 | 70 | 0 | Elsewhere |
| 44 | Ruthenium | Ru | 61 | 100 | 90 | 100 | United States and elsewhere |
| 45 | Rhodium | Rh | 3 | 100 | 40 | 100 | United States and elsewhere |
| 46 | Palladium | Pd | 6 | 97 | 40 | 45 | United States and elsewhere |
| 47 | Silver | Ag | 28 | 71 | 65 | 71 | Not critical |
| 48 | Cadmium | Cd | 16 | 100 | 77 | 25 | Not critical |
| 49 | Indium | In | 20 | 100 | 95 | 100 | United States and elsewhere |
| 50 | Tin | Sn | 74 | 3 | 70 | 75 | United States and elsewhere |
| 51 | Antimony | Sb | 14 | 80 | 95 | 85 | United States and elsewhere |
| 52 | Tellurium | Te | 85 | 100 | 99 | 90 | United States and elsewhere |
| 55 | Cesium | Cs | 0 | 100 | 33 | 100 | United States and elsewhere |
| 56 | Barium | Ba | 0 | 2 | 100 | 78 | United States and elsewhere |
| 57 | Lanthanum | La | 28 | 93 | 100 | 100 | United States and elsewhere |
| 58 | Cerium | Ce | 22 | 73 | 100 | 100 | United States and elsewhere |
| 59 | Praseodymium | Pr | 61 | 100 | 99 | 100 | United States and elsewhere |
| 60 | Neodymium | Nd | 74 | 100 | 99 | 100 | United States and elsewhere |
| 62 | Samarium | Sm | 97 | 82 | 99 | 100 | United States and elsewhere |
| 63 | Europium | Eu | 0 | 100 | 100 | 100 | United States and elsewhere |
| 64 | Gadolinium | Gd | 4 | 100 | 99 | 100 | United States and elsewhere |
| 65 | Terbium | Tb | 44 | 100 | 99 | 100 | United States and elsewhere |
| 66 | Dysprosium | Dy | 100 | 100 | 100 | 100 | United States and elsewhere |
| 67 | Holmium | Ho | 5 | 100 | 99 | 100 | United States and elsewhere |
| 68 | Erbium | Er | 8 | 100 | 97 | 100 | United States and elsewhere |
| 69 | Thulium | Tm | 1 | 100 | 99 | 100 | United States and elsewhere |
| 70 | Ytterbium | Yb | 2 | 100 | 100 | 100 | United States and elsewhere |
| 71 | Lutetium | Lu | 0 | 100 | 100 | 100 | United States and elsewhere |
| 72 | Hafnium | Hf | 76 | 100 | 99 | 100 | United States and elsewhere |
| 73 | Tantalum | Ta | 42 | 28 | 80 | 100 | United States and elsewhere |
| 74 | Tungsten | W | 80 | 5 | 75 | 60 | United States and elsewhere |
| 75 | Rhenium | Re | 70 | 100 | 48 | 80 | United States and elsewhere |
| 76 | Osmium | Os | 20 | 100 | 100 | 100 | United States and elsewhere |
| 77 | Iridium | Ir | 35 | 100 | 75 | 95 | United States and elsewhere |
| 78 | Platinum | Pt | 6 | 16 | 45 | 68 | United States and elsewhere |
| 79 | Gold | Au | 39 | 14 | 10 | 1 | Not critical |
| 80 | Mercury | Hg | 59 | 35 | 56 | 90 | Not critical |
| 81 | Thallium | Tl | 5 | 99 | 100 | 100 | Elsewhere |
| 82 | Lead | Pb | 2 | 10 | 8 | 31 | Not critical |
| 83 | Bismuth | Bi | 38 | 90 | 99 | 96 | United States and elsewhere |
| 92 | Uranium | U | 0 | 20 | 100 | 95 | United States and elsewhere |

The information of Supplementary Table 4 is displayed in the Supplementary Figures 7-9 in form of periodic tables.


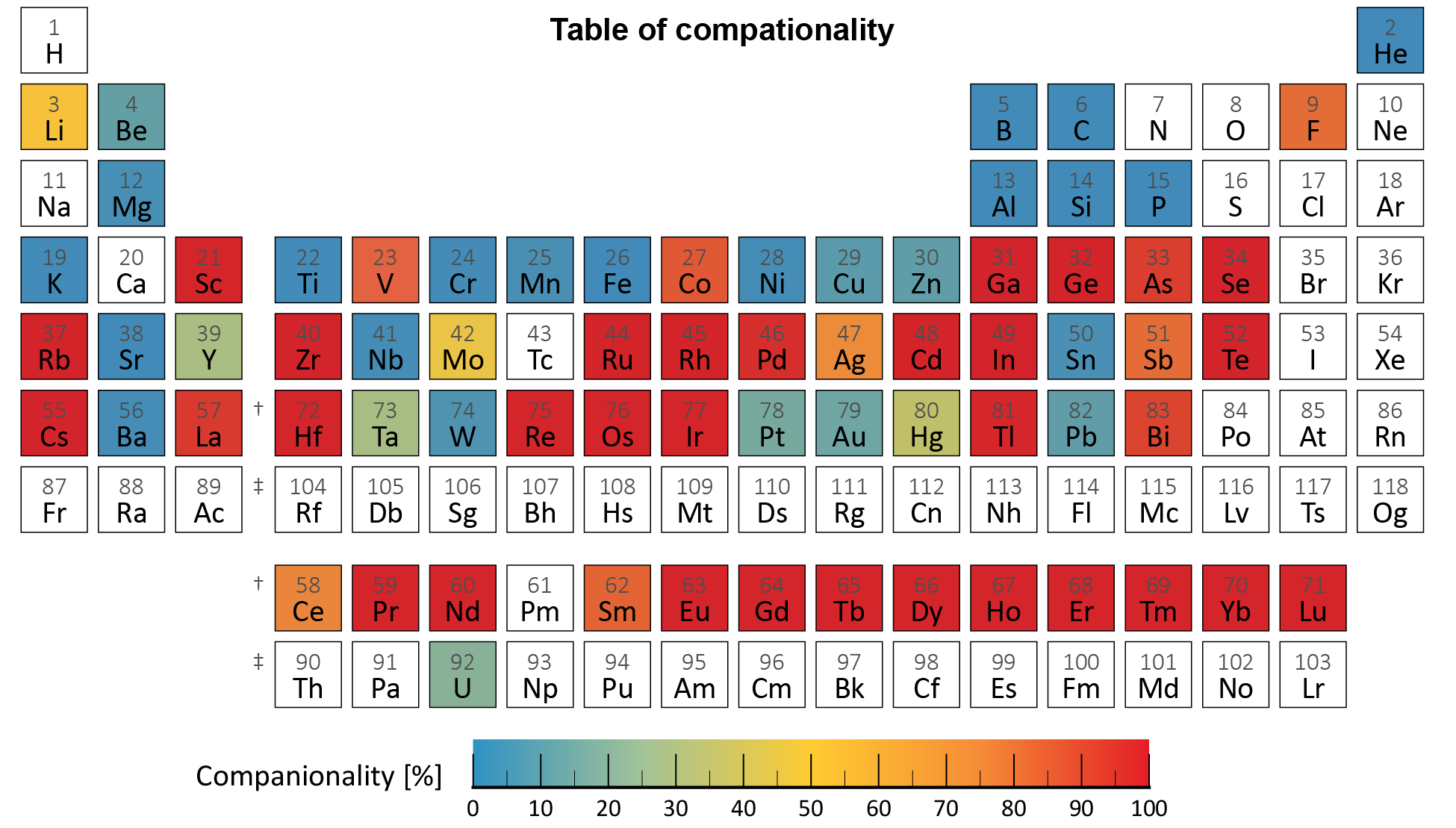


Supplementary Figure 7 – Periodic table of the companionality of elements. Adapted from Nassar et al. (2015) ^8^.


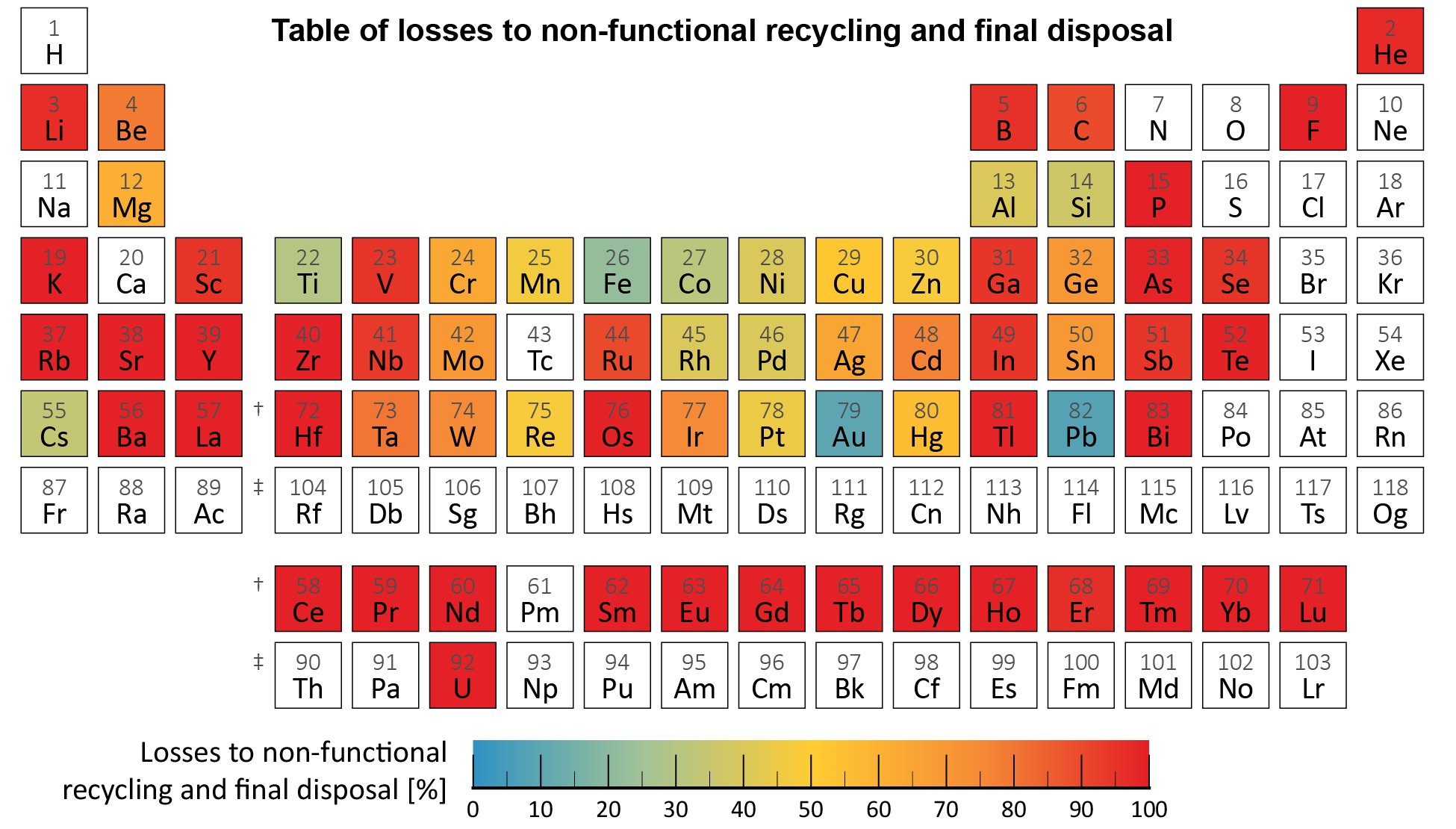


Supplementary Figure 8 – Periodic table of elements of the losses to non-functional recycling and final disposal. Adapted from UNEP (2011) ^43^.


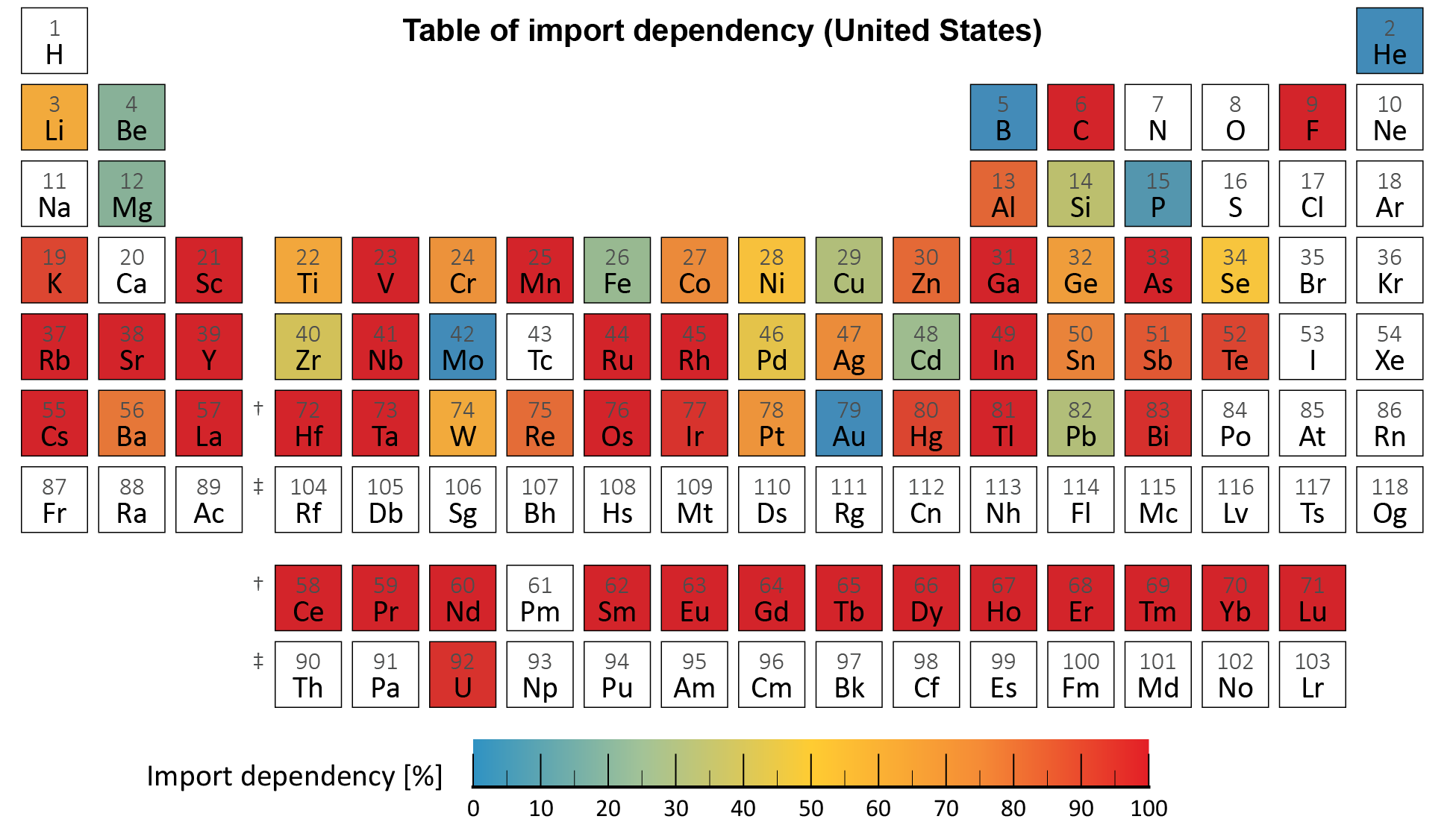


Supplementary Figure 9 – Periodic table of elements of the U.S. import dependency. Adapted from USGS Mineral Commodity Summaries (2018-2021) ^9, 12, 14, 15^.

# Supplementary References

1. Australian Government, 2020. Australian Critical Minerals Prospectus 2020. <https://www.austrade.gov.au/ArticleDocuments/5572/Australian_Critical_Minerals_Prospectus.pdf.aspx>. (Accessed 02 July 2021).

2. European Commission. *Study on the review of the list of Critical Raw Materials*. European Commission (2017).

3. Government of Canada, 2021. Canada’s Critical Minerals List 2021. <https://www.nrcan.gc.ca/sites/nrcan/files/mineralsmetals/pdf/Critical_Minerals_List_2021-EN.pdf>. (Accessed 02 July 2021).

4. Nakano J, 2021. The Geopolitics of Critical Minerals Supply Chains. <https://www.csis.org/analysis/geopolitics-critical-minerals-supply-chains>. (Accessed 02 July 2021).

5. U.S. Department of the Interior, 2018. Final List of Critical Minerals 2018. <https://www.federalregister.gov/d/2018-10667>.

6. Rudnick RL, Gao S. Composition of the continental crust. In: *Treatise on geochemistry*)<https://www.geol.umd.edu/~rudnick/PDF/Rudnick_Gao_Treatise.pdf>. (2003).

7. Emsley J. *Nature's building blocks: an A-Z guide to the elements*. Oxford University Press (2011).

8. Nassar NT, Graedel TE, Harper EM. By-product metals are technologically essential but have problematic supply. *Science Advances* **1**, e1400180 (2015).

9. U.S. Geological Survey, 2018. Mineral Commodity Summaries 2018.

10. Miatto A, Reck BK, West J, Graedel TE. The rise and fall of American lithium. *Resources, Conservation and Recycling* **162**, 105034 (2020).

11. Sun X, Hao H, Zhao F, Liu Z. Tracing global lithium flow: A trade-linked material flow analysis. *Resources, Conservation and Recycling* **124**, 50-61 (2017).

12. U.S. Geological Survey, 2021. Mineral Commodity Summaries 2021. <https://pubs.usgs.gov/periodicals/mcs2021/mcs2021.pdf>. (Accessed 15 March 2021).

13. European Commission. *Study on the review of the list of critical raw materials—Critical raw materials factsheets*. European Commission (2017).

14. U.S. Geological Survey, 2020. Mineral Commodity Summaries 2020.

15. U.S. Geological Survey. *Mineral Commodity Summaries 2017* (2017).

16. Jara AD, Betemariam A, Woldetinsae G, Kim JY. Purification, application and current market trend of natural graphite: A review. *International Journal of Mining Science and Technology* **29**, 671-689 (2019).

17. Roskill Information Services, 2013. Magnesium Metal: Global Industry Market & Outlook.

18. Mordor Intelligence, 2021. Scandium Market - Growth, Trends, Covid-19 Impact, And Forecasts (2021 - 2026). <https://www.mordorintelligence.com/industry-reports/scandium-market>.

19. Bedinger GM, 2021. Titanium [advanced release] 2017. <https://prd-wret.s3.us-west-2.amazonaws.com/assets/palladium/production/atoms/files/myb1-2017-titan.pdf>.

20. Reck BK, Rotter VS. Comparing Growth Rates of Nickel and Stainless Steel Use in the Early 2000s. *Journal of Industrial Ecology* **16**, 518-528 (2012).

21. Graedel TE, Harper EM, Nassar NT, Reck BK. On the materials basis of modern society. *Proceedings of the National Academy of Sciences* **112**, 6295-6300 (2015).

22. Copper Development Association, 2020. Annual Data 2020. <https://www.copper.org/resources/market_data/pdfs/annual-data-book-2020_final.pdf>. (Accessed 02 July 2021).

23. Licht C, Peiró LT, Villalba G. Global Substance Flow Analysis of Gallium, Germanium, and Indium: Quantification of Extraction, Uses, and Dissipative Losses within their Anthropogenic Cycles. *Journal of Industrial Ecology* **19**, 890-903 (2015).

24. Moskalyk RR. Review of germanium processing worldwide. *Minerals Engineering* **17**, 393-402 (2004).

25. Guyonnet D*, et al.* Material flow analysis applied to rare earth elements in Europe. *Journal of Cleaner Production* **107**, 215-228 (2015).

26. International Molybdenum Association, 2020. Uses of new Molybdenum. <https://www.imoa.info/molybdenum-uses/molybdenum-uses.php>. (Accessed 02 July 2021).

27. International Cadmium Association, 2019. Cadmium Applications. <https://www.cadmium.org/cadmium-applications>. (Accessed 02 July 2021).

28. International Tin Association, 2018. ITA Survey shows weaker tin use growth in 2018. <https://www.internationaltin.org/ita-survey-weaker-tin-use-growth-2018/>. (Accessed 02 July 2021).

29. Grand View Research, 2017. Europium Market Analysis By Application (Permanent Magnets, Catalysts, Glass Polishing, Phosphors, Ceramics, Metal Alloys, Glass Additives), By End-use, And Segment Forecasts, 2018 - 2025. <https://www.grandviewresearch.com/industry-analysis/europium-market>. (Accessed 02 July 2021).

30. Gambogi J, 2021. Rare Earths 2017 [Advance Release]. <https://prd-wret.s3.us-west-2.amazonaws.com/assets/palladium/production/atoms/files/myb1-2017-raree.pdf>.

31. Avalon Advanced Materials Inc., 2021. Erbium. <https://www.avalonadvancedmaterials.com/rare_metals/erbium/>. (Accessed 02 July 2021).

32. Thulium is a glass act. *NPG Asia Materials*, (2008).

33. AZO Materials, 2012. Ytterbium (Yb)-Discovery, Occurrence, Production, Properties and Applications of Ytterbium. <https://www.azom.com/article.aspx?ArticleID=7950>. (Accessed 02 July 2021).

34. AZO Materials, 2012. Lutetium (Lu) - Discovery, Occurrence, Production, Properties and Applications of Lutetium. <https://www.azom.com/article.aspx?ArticleID=7921>. (Accessed 02 July 2021).

35. Roskill Information Services, 2013. Niobium and Tantalum Factsheet. <https://prometia.eu/wp-content/uploads/2021/01/NIOBIUM-TANTALUM-v02.pdf>. (Accessed 02 July 2021).

36. MSP REFRAM, 2020. Tungsten Factsheet. <https://prometia.eu/wp-content/uploads/2021/01/TUNGSTEN.pdf>. (Accessed 02 July 2021).

37. MSP REFRAM, 2020. Rhenium Factsheet. <https://prometia.eu/wp-content/uploads/2021/01/RHENIUM.pdf>. (Accessed 02 July 2021).

38. Johnson Matthey, 2021. Pgm market report. <http://www.platinum.matthey.com/documents/new-item/pgm-market-reports/pgm-market-report-may-21.pdf>. (Accessed 02 July 2021).

39. S&P Global, 2021. Iridium hits all-time high of $6,000/oz on supply issues, strong demand. <https://www.spglobal.com/platts/en/market-insights/latest-news/metals/031921-iridium-hits-all-time-high-of-6000oz-on-supply-issues-strong-demand>. (Accessed 02 July 2021).

40. International Lead and Zinc Study Group, 2021. End Uses of Lead. <https://www.ilzsg.org/static/enduses.aspx?from=1>. (Accessed 02 July 2021).

41. NRCan, 2021. Distribution of lead consumption worldwide in 2019, by end use [Graph]. <https://www.statista.com/statistics/891778/distribution-of-global-lead-consumption-by-end-use/>. (Accessed 02 July 2021).

42. Chemistry RSo, 2021. Uranium. <https://www.rsc.org/periodic-table/element/92/uranium>. (Accessed 02 July 2021).

43. UNEP, 2011. Recycling rates of metals: A status report, A Report of the Working Group on the Global Metal Flows to the International Resource Panel. <http://hdl.handle.net/20.500.11822/8702>.
